# Metformin Inhibits Ferroptotic Cell Death by Suppressing Cysteine Deprivation‐Induced Mitochondrial Dysfunction

**DOI:** 10.1002/prp2.70330

**Published:** 2026-10-07

**Authors:** Se‐Kyeong Jang, Selim Kim, Gyeongmi Kim, Jungil Hong, In‐Chul Park, Hyeon‐Ok Jin

**Affiliations:** ^1^ Division of Radiation Biomedical Research Korea Institute of Radiological and Medical Sciences Seoul Republic of Korea; ^2^ Department of Food Science and Technology, College of Science and Convergence Technology Seoul Women's University Seoul Republic of Korea; ^3^ KIRAMS Radiation Biobank Korea Institute of Radiological and Medical Sciences Seoul Republic of Korea

**Keywords:** cysteine deprivation, ferroptosis, metformin, mitochondria

## Abstract

Ferroptosis, an iron‐dependent form of regulated cell death driven by excessive lipid peroxidation, has emerged as a promising therapeutic strategy for cancer. Metformin, a widely used biguanide antidiabetic drug, has recently been reported to induce ferroptosis and exert anticancer effects in various malignancies. However, metformin or its analogue phenformin did not induce ferroptotic cell death in H1299 non‐small cell lung cancer cells and HEYA8 ovarian cancer cells for up to 24 h. Instead, both drugs attenuated cysteine deprivation‐induced ferroptotic cell death, which also appeared to involve necroptotic signaling. This effect was accompanied by reductions in reactive oxygen species generation, iron accumulation, and lipid peroxidation at both the cellular and mitochondrial levels. Notably, metformin and phenformin suppressed cysteine deprivation‐induced mitochondrial membrane hyperpolarization and increased oxygen consumption, suggesting suppression of mitochondrial bioenergetic activity associated with cysteine deprivation‐induced ferroptosis. Cysteine deprivation markedly reduced glutathione (GSH) levels and glutathione peroxidase 4 (GPX4) expression. Although metformin alone also reduced intracellular GSH levels, metformin did not restore GSH levels or GPX4 expression under cysteine deprivation despite suppressing ferroptosis, indicating that its anti‐ferroptotic effects are independent of the canonical GSH‐GPX4 axis. Collectively, these findings underscore that the use of biguanides should be carefully considered when applying cysteine deprivation‐based ferroptosis‐inducing strategies in cancer.

## 1 Introduction

Ferroptosis is an iron‐dependent form of regulated cell death characterized by extensive membrane damage resulting from lipid peroxidation [1, 2]. This process is driven by excess ferrous ions, reactive oxygen species (ROS), and phospholipids containing polyunsaturated fatty acids [3]. Ferroptosis can be triggered through two major pathways: the transporter‐dependent pathway and enzyme‐regulated pathway [4]. The transporter‐dependent pathway is initiated by the activation of the iron transporters or by inhibition of the cystine/glutamate antiporter system SLC7A11/xCT, whereas the enzyme‐regulated pathway is mainly induced by suppression of the expression or activity of intracellular antioxidant enzymes, such as glutathione peroxidase 4 (GPX4) [5].

Cancer cells exhibit elevated iron requirements and increased ROS levels, rendering them more susceptible to ferroptosis compared with normal cells [3, 6]. This vulnerability has positioned ferroptosis as a promising therapeutic strategy for cancers characterized by aberrant lipid metabolism and resistance to conventional therapies [3, 7]. The interplay between ferroptosis and lipid metabolism has been linked to cancer initiation, progression, invasion, metastasis, and therapy resistance [8]. Pharmacological modulators of lipid peroxidation have shown efficacy in suppressing multidrug‐resistant cancer cells [9], and ferroptosis inducers can overcome resistance to chemotherapeutic agents such as cisplatin, docetaxel, and sorafenib [10, 11].

Cysteine is a critical determinant of ferroptosis susceptibility. Its depletion, either through culture media restriction or pharmacological inhibition of SLC7A11/xCT transporter, strongly induces ferroptosis in cancer cells [12, 13]. In this study, cysteine deprivation specifically refers to the use of cysteine‐free medium and is distinct from pharmacological inhibition of system Xc^−^‐mediated cystine uptake by agents such as imidazole ketone erastin (IKE). This effect is largely attributable to the disruption of cysteine‐dependent metabolic pathways, including glutathione (GSH) synthesis, coenzyme A synthesis, and iron–sulfur biogenesis [14, 15].

Metformin is a widely used biguanide that is considered a first‐line therapy for Type 2 diabetes mellitus and is generally well tolerated, associated with minimal side effects, and inexpensive [16]. Recently, there has been growing interest in repurposing metformin for cancer therapy, particularly in combination regimens [17, 18]. Emerging evidence suggests that metformin can induce or enhance ferroptosis in several cancer types through diverse mechanisms, including inhibition of the NRF2/HO‐1 signaling pathway in lung cancer cells [19], suppression of GPX4 through upregulation of miR‐324‐3p and destabilization of SLC7A11 in breast cancer cells [20, 21], and sensitization to sorafenib‐induced ferroptosis through inhibition of the ATF4/STAT3 pathway in hepatocellular carcinoma cells [22].

In the present study, we investigated the effects of metformin and its analogue phenformin on cysteine deprivation‐induced ferroptosis in cancer cells and explored the underlying mechanisms. Unexpectedly, both drugs attenuated cysteine deprivation‐induced ferroptotic cell death despite GSH depletion and GPX4 downregulation. Metformin and phenformin suppressed mitochondrial membrane hyperpolarization and increased oxygen consumption induced by cysteine deprivation, implicating mitochondrial alterations in their inhibitory effects on ferroptosis. These findings suggest that the use of biguanides should be carefully considered when developing therapeutic strategies aimed at inducing ferroptosis through cysteine deprivation.

## 2 Materials and Methods

### 2.1 Cell Culture, Cysteine Deprivation, and Reagents

H1299 human non‐small cell lung cancer cells (RRID: CVCL_0060) were obtained from the American Type Culture Collection (ATCC; Manassas, VA, USA). HEYA8 human ovarian cancer cells were provided by Dr. Anil K. Sood (The University of Texas MD Anderson Cancer Center, Houston, TX, USA). H1299 and HEYA8 cells were cultured in Roswell Park Memorial Institute (RPMI) 1640 medium (#LM011‐01; Welgene, Gyeongsangbuk‐do, Republic of Korea) supplemented with 10% fetal bovine serum (FBS; #16000–044; Gibco, Thermo Fisher Scientific, Waltham, MA, USA). The cell lines were routinely tested for mycoplasma contamination. Custom‐made cysteine‐free RPMI 1640 medium (#LM011‐136; Welgene) was supplemented with 10% dialyzed FBS (#26400–044; Gibco, Thermo Fisher Scientific), from which low‐molecular‐weight components had been removed. Metformin (#317240), phenformin (#P7045), thiazolyl blue tetrazolium bromide (MTT; #M2128), and rotenone (#R8875) were purchased from Sigma‐Aldrich (Merck KGaA, Darmstadt, Germany). Carbonyl cyanide m‐chlorophenyl hydrazone (CCCP; #M34152) was purchased from Thermo Fisher Scientific. Deferoxamine mesylate (#S5742), ferrostatin‐1 (#S7243), liproxstatin‐1 (#S7699), necrostatin‐1 (#S8037), z‐VAD‐fmk (#S7023), and cyclosporine A (#S2286) were purchased from Selleck Chemicals (Houston, TX, USA).

### 2.2 Cell Viability Assay

Cell viability was determined using the MTT assay. Cells at approximately 40%–50% confluence were treated with metformin or phenformin under cysteine‐deprived conditions. MTT reagent was then added at a final concentration of 0.5 mg/mL, and the resulting purple formazan crystals were dissolved in isopropanol. Absorbance was measured at 570 nm, and cell viability was expressed as a percentage of the control.

### 2.3 Cell Death Assay

Cell death was assessed by detecting dead cells using propidium iodide (PI, #V13242, Invitrogen; Thermo Fisher Scientific). After treating cysteine‐deprived cells with metformin or phenformin, cell pellets were collected in cold PBS and stained with PI at a concentration of 1 μg/mL. A total of 5000 cells were analyzed using a CytoFLEX flow cytometer (#A00‐1‐1102, Beckman Coulter, Brea, CA, USA) with a 488 nm blue laser and a 585/42 nm bandpass filter for PI detection.

### 2.4 Lipid Peroxidation and Mitochondrial Lipid Peroxidation Analysis

Lipid peroxidation and mitochondrial lipid peroxidation were assessed using the C11‐BODIPY 581/591 (#D3861, Invitrogen; Thermo Fisher Scientific) and MitoPeDPP (#466, Dojindo, Kumamoto, Japan), respectively. Cells cultured under cysteine‐deprived conditions were treated with metformin or phenformin, followed by staining with 2 μM C11‐BODIPY 581/591 at 37°C for 30 min or 100 nM MitoPeDPP at 37°C for 20 min. The stained cells were then collected in cold PBS, and 5000 cells were analyzed using a CytoFLEX flow cytometer (Beckman Coulter). FITC fluorescence was detected using a 488 nm blue laser and a 525/40 nm bandpass filter.

### 2.5 Reactive Oxygen Species (ROS) and Mitochondrial ROS Analysis

Intracellular ROS and mitochondrial ROS were measured using 2′,7′‐dichlorofluorescein diacetate (DCFH‐DA, #35845, Sigma‐Aldrich; Merck KGaA) and MitoSOX (#M36008, Invitrogen; Thermo Fisher Scientific), respectively. Cells cultured under cysteine‐deprived conditions were treated with metformin or phenformin, followed by staining with 100 nM DCFH‐DA or 2.5 μM MitoSOX at 37°C for 30 min. The stained cells were then collected in cold PBS, and 5000 cells were analyzed using a CytoFLEX flow cytometer (Beckman Coulter). Intracellular ROS was detected as FITC fluorescence using a 488 nm blue laser and a 525/40 nm bandpass filter, whereas mitochondrial ROS was detected as PE fluorescence using a 488 nm blue laser and a 585/42 nm bandpass filter.

### 2.6 Ferrous Ion (Fe^2+^) and Mitochondrial Fe^2+^ Assay

Intracellular Fe^2+^ and mitochondrial Fe^2+^ were detected using FerroOrange (#F374, Dojindo) and Mito‐FerroGreen (#M489, Dojindo), respectively. Cells cultured under cysteine‐deprived conditions were treated with metformin or phenformin. For intracellular Fe^2+^ detection, the cells were collected and stained with 1 μM FerroOrange at 37°C for 30 min, followed by collection in cold PBS. For mitochondrial Fe^2+^ detection, the cells were stained with 5 μM Mito‐FerroGreen at 37°C for 30 min and subsequently collected in cold PBS. A total of 5000 cells were analyzed using a CytoFLEX flow cytometer (Beckman Coulter). Intracellular Fe^2+^ was detected as PE fluorescence using a 488 nm blue laser and a 585/42 nm bandpass filter, whereas mitochondrial Fe^2+^ was detected as FITC fluorescence using a 488 nm blue laser and a 525/40 nm bandpass filter.

### 2.7 Reduced Glutathione (GSH) and Oxidized Glutathione (GSSG) Assay

Intracellular levels of GSH and GSSG were measured using a commercially available GSH/GSSG ratio detection assay kit (#ab138881, Abcam, Cambridge, UK) according to the manufacturer's instructions. Fluorescence was measured using a SpectraMax i3x microplate reader (Molecular Devices, San Jose, CA, USA) at excitation and emission wavelengths of 485 and 535 nm, respectively.

### 2.8 Mitochondrial Membrane Potential (MMP) Assay

MMP was detected by tetramethylrhodamine ethyl ester perchlorate (TMRE, #HY‐D0985A, MedChemExpress, Monmouth Junction, NJ, USA). After treating cysteine‐deprived cells with metformin or phenformin, cells were washed with Hank's balanced salt solution and then incubated with 100 nM TMRE at 37°C for 15 min. A total of 5000 cells were analyzed using a CytoFLEX flow cytometer (Beckman Coulter) with a 488 nm blue laser and a 585/42 nm bandpass filter for PE detection.

### 2.9 Oxygen Consumption Assay

Oxygen consumption was measured using the extracellular oxygen consumption rate plate assay kit (#E297‐12, Dojindo) according to the manufacturer's instructions. Briefly, cells were seeded in 96‐well clear‐bottom black plates and exposed to cysteine‐deprived conditions in the presence of metformin or phenformin. Following the addition of the oxygen probe, the wells were immediately sealed with mineral oil. Fluorescence was recorded at 380/650 nm (Ex/Em) every 1.5 min for a total duration of 180 min using a SpectraMax i3x microplate reader (Molecular Devices, Sunnyvale, CA, USA). Fluorescence intensity was normalized to the blank control using culture medium.

### 2.10 Western Blot Analysis

Protein expression levels were determined by Western blot analysis as described previously [23]. Primary antibodies against glutathione peroxidase 4 (ab125066, Abcam, Cambridge, UK) and β‐actin (#A5316, Sigma‐Aldrich; Merck KGaA) were used. The quantified levels of protein expression were assessed using ImageJ software (version 1.52a; National Institutes of Health, Bethesda, MD, USA).

### 2.11 Statistical Analysis

Each experiment was independently performed three times on different experimental days using separately prepared cell cultures, and results are presented as the mean ± standard deviation (SD). Statistical analyses were performed using Student's *t*‐test for comparisons between two groups, or one‐way ANOVA followed by Tukey's post hoc test for comparisons among three or more groups, using GraphPad Prism software (version 9.0; GraphPad Software, San Diego, CA, USA). A *p*‐value < 0.05 was considered statistically significant.

## 3 Results

### 3.1 Metformin and Phenformin Attenuate Cysteine Deprivation‐Induced Cell Death

We first investigated the effects of the antidiabetic drugs metformin and phenformin on cysteine deprivation‐induced cell death in H1299 non‐small cell lung cancer (NSCLC) cells. Cells were cultured for 24 h in complete or cysteine‐deprived medium in the presence of various concentrations of metformin or phenformin. MTT assays showed that treatment with metformin or phenformin alone decreased cell viability in a dose‐dependent manner; however, cell viability remained above approximately 75% even after treatment with up to 20 mM metformin or 200 μM phenformin (Figure 1A,B). Under cysteine‐deprived conditions, both drugs dose‐dependently attenuated the reduction in cell viability in H1299 cells (Figure 1A,B). Similarly, metformin and phenformin attenuated the cysteine deprivation‐induced reduction in cell viability in HEYA8 ovarian cancer cells (Figure 1C,D). Propidium iodide staining showed that treatment with metformin or phenformin alone induced less than 10% cell death, whereas both drugs markedly reduced cysteine deprivation‐induced cell death in H1299 cells (Figure 1E,F). Taken together, these findings indicate that metformin and phenformin attenuate cysteine deprivation‐induced cell death in H1299 NSCLC and HEYA8 ovarian cancer cells.

**FIGURE 1:**
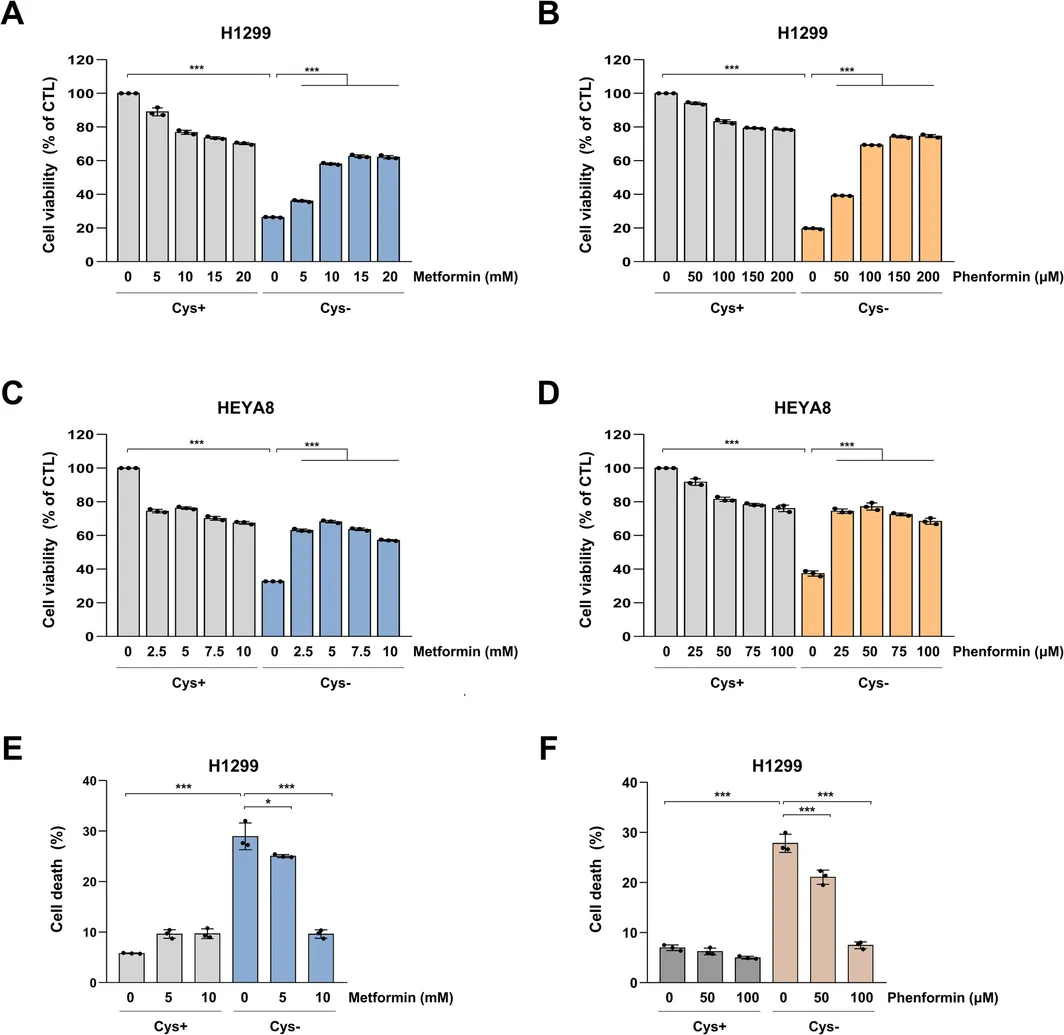
Effects of metformin and phenformin on cysteine deprivation‐induced cell death. (A–D) H1299 and HEYA8 cells were treated with the indicated concentrations of metformin or phenformin for 24 h in complete or cysteine‐deprived medium. Cell viability was assessed by MTT assay and is presented as a percentage relative to the control. (E, F) H1299 cells were treated with the indicated concentrations of metformin or phenformin for 24 h in complete or cysteine‐deprived medium. Cell death was evaluated by flow cytometry based on the percentage of PI‐positive cells. Data are presented as the mean ± SD (*n* = 3; *p < 0.05, ***p < 0.001). CTL, control; PI, propidium iodide; SD, standard deviation.

### 3.2 Metformin and Phenformin Attenuate Cysteine Deprivation‐Induced Ferroptotic Cell Death

To better characterize the cell death attenuated by metformin and phenformin, we first examined the type of cell death induced by cysteine deprivation using pharmacological inhibitors in H1299 and HEYA8 cells. Under cysteine‐deprived conditions, the ferroptosis inhibitors ferrostatin‐1 (Fer‐1) and liproxstatin‐1 (Lip‐1) markedly restored cell viability in both cell lines (Figure 2A,C). The iron chelator deferoxamine (DFO) likewise markedly restored cell viability in H1299 and HEYA8 cells (Figure 2B,D). In contrast, the apoptosis inhibitor z‐VAD‐fmk (z‐VAD) had no significant effect on cell viability (Figure 2A,C). Interestingly, the necroptosis inhibitor necrostatin‐1 (Nec‐1) also markedly restored cell viability in both cell lines (Figure 2A,C). We next examined the effects of these inhibitors on cysteine deprivation‐induced lipid peroxidation using C11‐BODIPY 581/591. Fer‐1, Lip‐1, and DFO markedly reduced lipid peroxidation in both H1299 and HEYA8 cells, whereas z‐VAD had no significant effect (Figure 2E–H). Interestingly, Nec‐1 also significantly reduced cysteine deprivation‐induced lipid peroxidation in both cell lines (Figure 2E,G). Collectively, these findings suggest that cysteine deprivation‐induced cell death is primarily mediated by ferroptosis but also involves necroptotic signaling.

**FIGURE 2:**
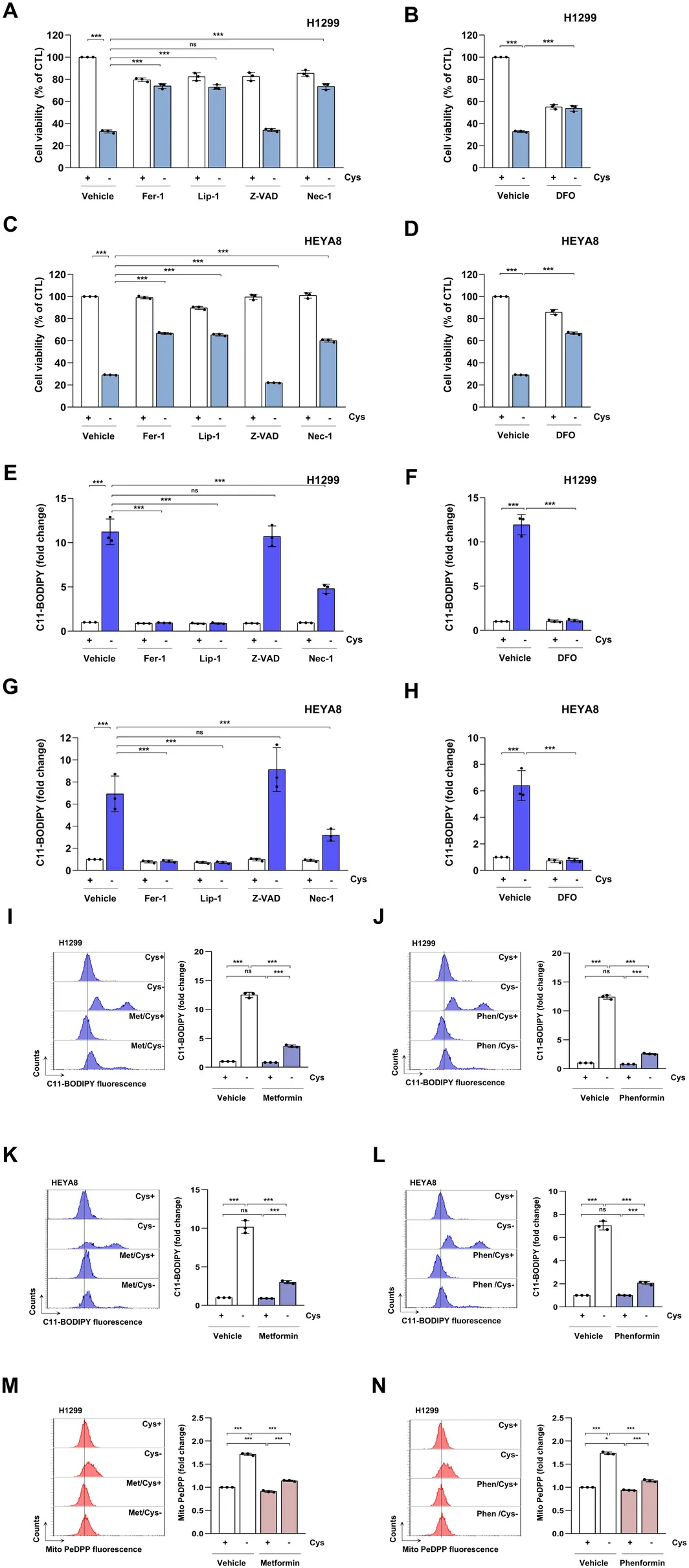
Metformin and phenformin suppress cysteine deprivation‐induced lipid peroxidation. (A–H) H1299 and HEYA8 cells were cultured in complete or cysteine‐deprived medium in the presence of the indicated inhibitors for 24 h (A–D) or 12 h (E–H). (A–D) Cell viability was assessed by MTT assay. (I–N) H1299 and HEYA8 cells were treated with 10 mM metformin or 100 μM phenformin for 12 h in complete or cysteine‐deprived medium. (E–L) Lipid peroxidation was assessed by flow cytometry using C11‐BODIPY. (M, N) Mitochondrial lipid peroxidation was assessed using MitoPeDPP. In panels I–N, left panels show representative histograms, and right panels show bar graphs of the MFI relative to the control. Data are presented as the mean ± SD (*n* = 3; *p < 0.05, ***p < 0.001; ns, not significant). DFO, deferoxamine; Fer‐1, ferrostatin‐1; Lip‐1, liproxstatin‐1; MFI, mean fluorescence intensity; Nec‐1, necrostatin‐1; SD, standard deviation; z‐VAD, z‐VAD‐fmk.

We then examined the effects of metformin and phenformin on cysteine deprivation‐induced lipid peroxidation. Both drugs markedly reduced cysteine deprivation‐induced lipid peroxidation in H1299 and HEYA8 cells (Figure 2I–L). MitoPeDPP analysis further showed that both drugs markedly attenuated cysteine deprivation‐induced mitochondrial lipid peroxidation in H1299 cells (Figure 2M,N). Taken together, these findings support the inhibitory effects of metformin and phenformin on cysteine deprivation‐induced ferroptosis.

### 3.3 Metformin and Phenformin Attenuate Cysteine Deprivation‐Induced Reactive Oxygen Species (ROS) Production

Given that ROS promote lipid peroxidation and ferroptotic cell death [24, 25], we next investigated whether metformin and phenformin suppress cysteine deprivation‐induced ROS accumulation. Cysteine deprivation markedly increased intracellular ROS levels, as assessed by DCF fluorescence, whereas treatment with metformin or phenformin alone did not significantly alter intracellular ROS levels (Figure 3A,B). Treatment with either metformin or phenformin significantly attenuated the increase in intracellular ROS induced by cysteine deprivation (Figure 3A,B). Mitochondrial superoxide levels, assessed using MitoSOX, were also markedly increased following cysteine deprivation, whereas treatment with metformin or phenformin alone slightly increased mitochondrial superoxide levels (Figure 3C,D). Both drugs significantly attenuated cysteine deprivation‐induced mitochondrial superoxide accumulation (Figure 3C,D).

**FIGURE 3:**
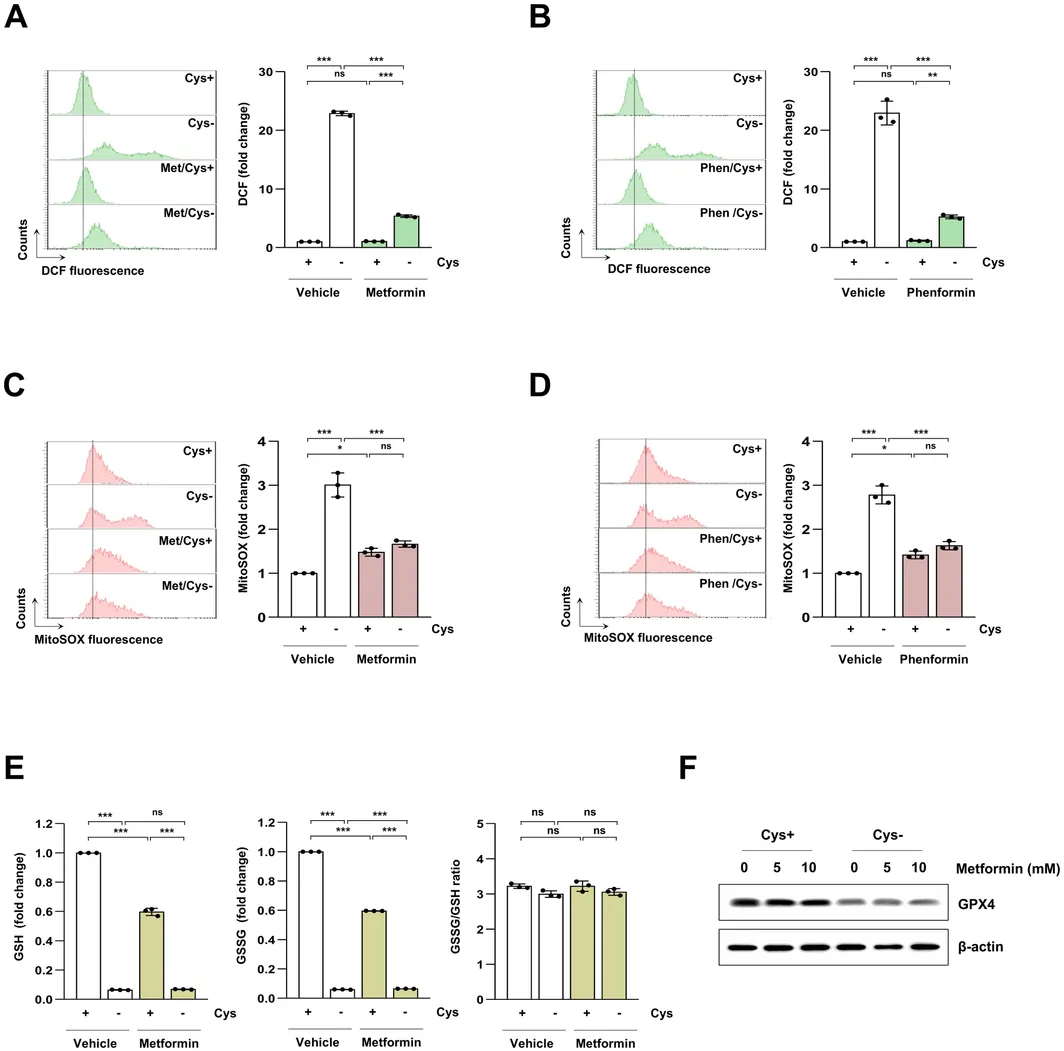
Metformin and phenformin suppress cysteine deprivation‐induced reactive oxygen species (ROS). (A–E) H1299 cells were treated with 10 mM metformin or 100 μM phenformin for 12 h in complete or cysteine‐deprived medium. (A, B) Intracellular ROS levels were assessed by flow cytometry using DCFH‐DA. (C, D) Mitochondrial superoxide levels were assessed using MitoSOX. Left panels show representative histograms, and right panels show bar graphs of the MFI relative to the control. (E) Intracellular GSH and GSSG levels were measured, and the GSSG/GSH ratio was calculated. (F) H1299 cells were treated with the indicated concentrations of metformin for 12 h in complete or cysteine‐deprived medium. GPX4 protein expression was analyzed by western blot analysis, with β‐actin used as a loading control. Data are presented as the mean ± SD (*n* = 3; *p < 0.05, ***p < 0.001; ns, not significant). GSH, reduced glutathione; GSSG, oxidized glutathione; MFI, mean fluorescence intensity; ROS, reactive oxygen species; SD, standard deviation.

Cysteine deprivation impairs glutathione (GSH) synthesis, thereby compromising GSH‐dependent glutathione peroxidase 4 (GPX4) function and promoting ferroptosis [1, 26]. Therefore, we investigated whether the inhibitory effects of metformin on cysteine deprivation‐induced ferroptosis involve the GSH‐GPX4 axis. Cysteine deprivation markedly decreased intracellular GSH and GSSG levels; however, the GSSG/GSH ratio was not significantly altered (Figure 3E). GPX4 protein expression was also markedly reduced (Figure 3F). Treatment with metformin alone decreased intracellular GSH and GSSG levels but did not significantly affect the GSSG/GSH ratio or GPX4 protein expression (Figure 3E,F). Under cysteine‐deprived conditions, metformin did not significantly alter intracellular GSH and GSSG levels, the GSSG/GSH ratio, or GPX4 protein expression (Figure 3E,F).

Taken together, these findings indicate that metformin attenuates cysteine deprivation‐induced ROS accumulation without restoring intracellular GSSG/GSH levels, the GSSG/GSH ratio or GPX4 protein expression, suggesting that its inhibitory effect is independent of the canonical GSH‐GPX4 axis.

### 3.4 Metformin and Phenformin Attenuate Cysteine Deprivation‐Induced Iron Accumulation

We next investigated whether metformin and phenformin could suppress cysteine deprivation‐induced iron accumulation. Staining with FerroOrange, a fluorescent probe for detecting ferrous ion (Fe^2+^), revealed that cysteine deprivation markedly increased intracellular Fe^2+^ levels, whereas metformin or phenformin alone did not alter intracellular Fe^2+^ levels (Figure 4A,B). Treatment with either metformin or phenformin significantly attenuated cysteine deprivation‐induced intracellular Fe^2+^ accumulation (Figure 4A,B).

**FIGURE 4:**
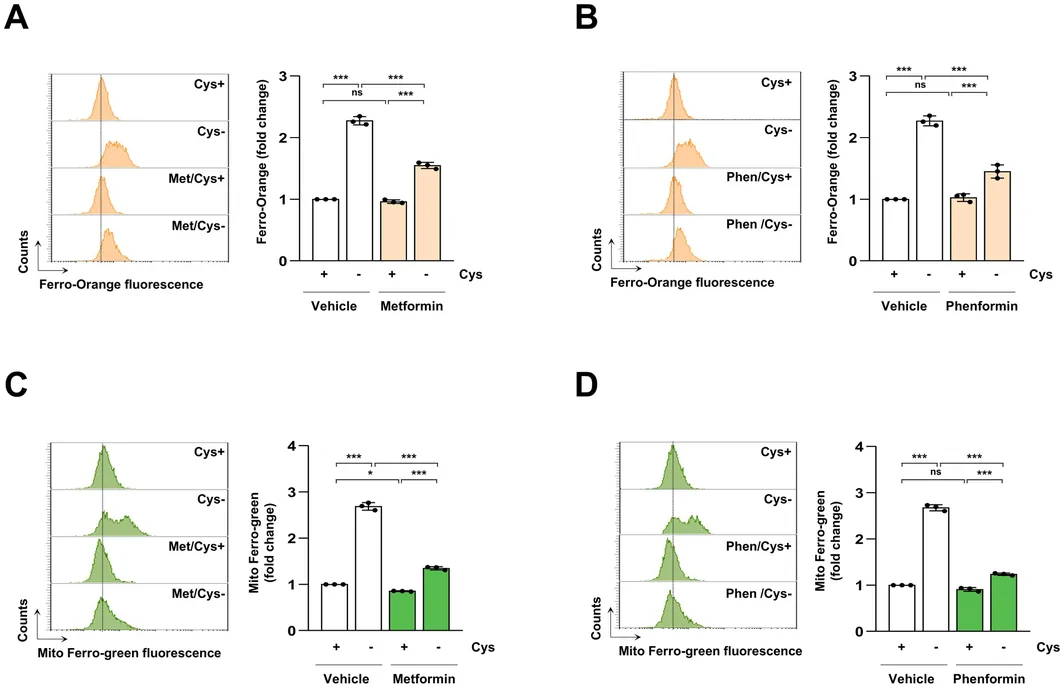
Metformin and phenformin suppress cysteine deprivation‐induced ferrous iron accumulation. (A–D) H1299 cells were treated with 10 mM metformin or 100 μM phenformin for 12 h in complete or cysteine‐deprived medium. (A, B) Intracellular Fe^2+^ levels were assessed by flow cytometry using FerroOrange. (C, D) Mitochondrial Fe^2+^ levels were assessed using Mito‐FerroGreen. Left panels show representative histograms, and right panels show bar graphs of the MFI relative to the control. Data are presented as the mean ± SD (*n* = 3; *p < 0.05, ***p < 0.001; ns, not significant). MFI, mean fluorescence intensity; SD, standard deviation.

Similarly, staining with Mito‐FerroGreen, a *mitochondria‐specific* Fe^2+^ *probe, showed that* cysteine deprivation markedly increased mitochondrial Fe^2+^ levels, whereas metformin or phenformin alone did not alter mitochondrial Fe^2+^ levels (Figure 4C,D). Both drugs significantly attenuated cysteine deprivation‐induced mitochondrial Fe^2+^ accumulation (Figure 4C,D). Taken together, these results indicate that metformin and phenformin suppress cysteine deprivation‐induced intracellular and mitochondrial Fe^2+^ accumulation.

### 3.5 Metformin and Phenformin Suppress Cysteine Deprivation‐Induced MMP Hyperpolarization and Increased Oxygen Consumption

Previous studies have reported that mitochondrial membrane potential (MMP) hyperpolarization and increased oxygen consumption induced by cysteine deprivation contribute to ferroptosis [2, 25]. Based on these observations, we next investigated whether metformin and phenformin modulate these mitochondrial alterations under cysteine deprivation. To assess early mitochondrial alterations before substantial cell loss could affect the measurements, MMP and oxygen consumption were evaluated at 2 h after cysteine deprivation. TMRE staining revealed that cysteine deprivation induced marked MMP hyperpolarization (Figure 5A,B). Oxygen consumption was also significantly increased under cysteine deprivation (Figure 5C,D). Treatment with metformin or phenformin alone markedly reduced MMP but only slightly increased oxygen consumption (Figure 5A–5D). Treatment with either drug significantly attenuated cysteine deprivation‐induced MMP hyperpolarization and the increase in oxygen consumption (Figure 5A–5D). Interestingly, to further determine whether the mitochondrial alterations induced by cysteine deprivation are associated with ferroptosis, we examined the effects of Fer‐1 on MMP and mitochondrial ROS. Fer‐1 attenuated the cysteine deprivation‐induced increase in MMP as well as mitochondrial ROS accumulation, suggesting that these mitochondrial alterations are associated with cysteine deprivation‐induced ferroptosis (Figure S1). Taken together, these findings indicate that metformin and phenformin attenuate cysteine deprivation‐induced MMP hyperpolarization and increased oxygen consumption.

**FIGURE 5:**
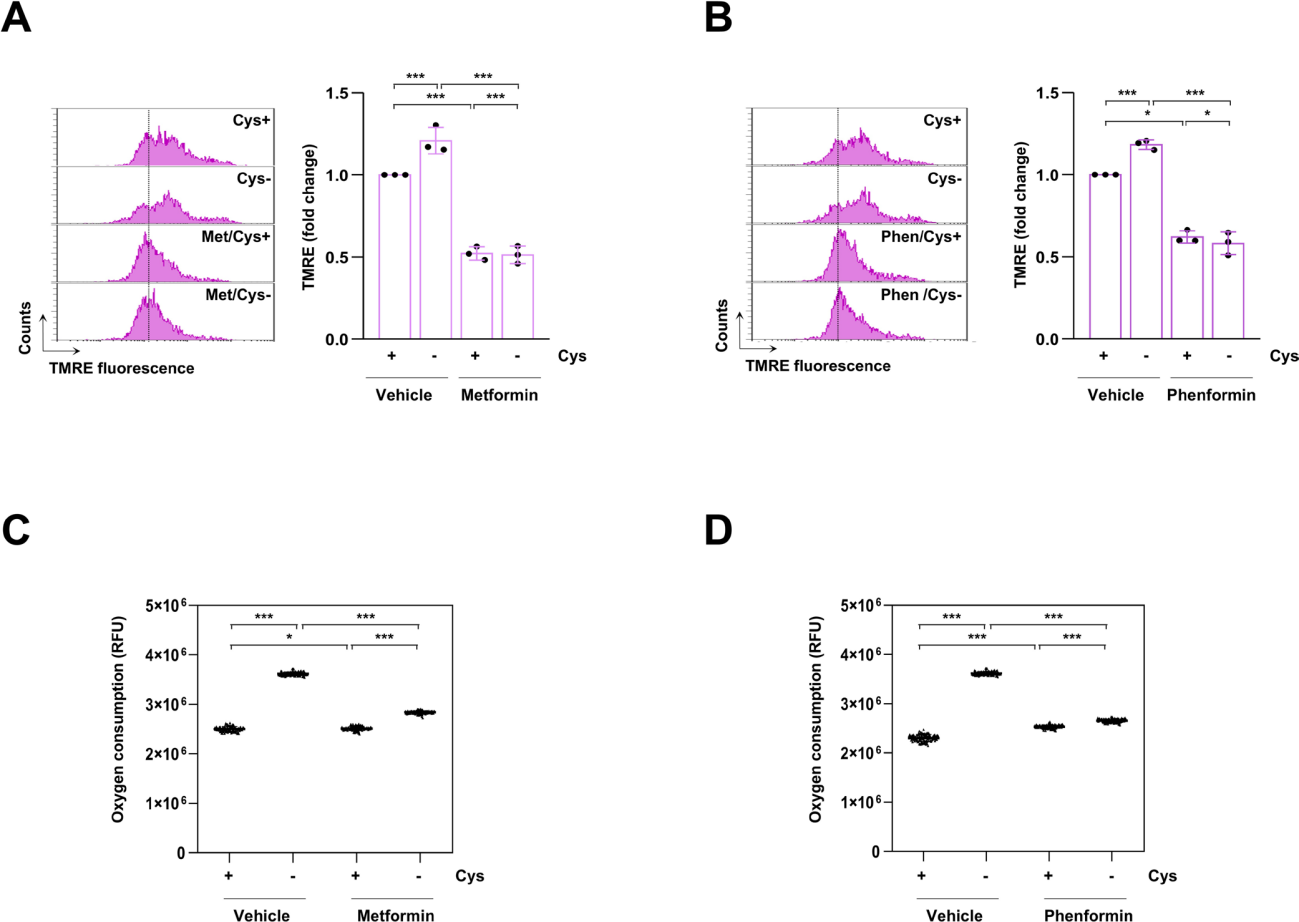
Metformin and phenformin attenuate cysteine deprivation‐induced mitochondrial hyperpolarization and increased oxygen consumption. (A–D) H1299 cells were treated with 10 mM metformin (A, C) or 100 μM phenformin (B, D) for 2 h in complete or cysteine‐deprived medium. (A, B) Mitochondrial membrane potential was assessed by flow cytometry using TMRE. Left panels show representative histograms, and right panels show bar graphs of the MFI relative to the control. (C, D) Oxygen consumption was measured using an extracellular oxygen consumption assay. Dots represent oxygen consumption values measured at 1.5‐min intervals over 180 min. Data are presented as the mean ± SD (*n* = 3; *p < 0.05, ***p < 0.001; ns, not significant). MFI, mean fluorescence intensity; SD, standard deviation; TMRE, tetramethylrhodamine ethyl ester perchlorate.

## 4 Discussion

Metformin, a widely prescribed biguanide and first‐line therapy for Type 2 diabetes mellitus, has been reported to induce ferroptosis as a single agent or to enhance ferroptosis in combination with anticancer therapies in several cancer models [19, 20, 21, 22, 27]. However, anti‐ferroptotic effects of metformin have also been described in various non‐malignant disease models, including diabetic osteoporosis and pancreatic β‐cell injury [28, 29], highlighting the context‐dependent role of metformin in the regulation of ferroptosis. In the present study, metformin and its analogue phenformin attenuated cysteine deprivation‐induced ferroptosis in both H1299 non‐small cell lung cancer (NSCLC) and HEYA8 ovarian cancer cells. A549, H460, H23, and H820 NSCLC cell lines exhibited substantially smaller reductions in cell viability following 24 h of cysteine deprivation, indicating lower sensitivity to cysteine deprivation‐induced ferroptosis (Figure S2). Consequently, metformin did not markedly alter cell viability under cysteine deprivation in these cell lines at any of the concentrations tested.

This finding is particularly noteworthy because metformin is a well‐established inhibitor of mitochondrial Complex I [30, 31], whereas cysteine deprivation‐induced ferroptosis critically depends on mitochondrial metabolic activity, including the tricarboxylic acid cycle and electron transport chain [2, 25]. Consistent with this concept, pharmacological inhibition of mitochondrial Complex I with rotenone suppresses cysteine deprivation‐induced ferroptosis [2, 25]. Similarly, metformin and phenformin significantly attenuated cysteine deprivation‐induced increases in oxygen consumption and mitochondrial membrane hyperpolarization, while reducing ROS production, iron accumulation, and lipid peroxidation at both the cellular and mitochondrial levels, ultimately suppressing ferroptotic cell death. Collectively, these findings suggest that suppression of mitochondrial Complex I‐mediated metabolic activity contributes, at least in part, to the inhibitory effects of metformin and phenformin on cysteine deprivation‐induced ferroptosis.

However, the concentrations of metformin required to suppress cysteine deprivation‐induced ferroptosis in our study were substantially higher than clinically achievable plasma concentrations (approximately 10–40 μM) [31, 32]. Indeed, metformin at micromolar concentrations did not detectably suppress cysteine deprivation‐induced ferroptosis in either H1299 or HEYA8 cells, whereas inhibitory effects became evident at millimolar concentrations. This concentration range is consistent with previous in vitro studies showing that the mitochondrial effects of metformin, including Complex I inhibition, generally require millimolar concentrations [31, 32]. Therefore, our findings should primarily be interpreted as mechanistic evidence that modulation of mitochondrial bioenergetic activity can suppress cysteine deprivation‐induced ferroptosis, rather than as direct evidence for the clinical applicability of metformin at therapeutic concentrations. Further studies will be required to determine whether similar mitochondrial modulation can be achieved under clinically relevant conditions.

Cysteine is a critical regulator of ferroptosis because it serves as a precursor for glutathione (GSH) synthesis and as an essential sulfur donor for iron–sulfur (Fe‐S) cluster biogenesis [33]. Metformin and phenformin significantly attenuated cysteine deprivation‐induced ROS accumulation without restoring intracellular GSH levels or glutathione peroxidase 4 (GPX4) expression, suggesting that their inhibitory effects against cysteine deprivation‐induced ferroptosis are independent of the canonical GSH‐GPX4 antioxidant system. A previous study has shown that cysteine deprivation impairs Fe‐S cluster biogenesis, thereby disrupting cellular iron homeostasis and increasing intracellular labile Fe^2+^ levels [33]. In the present study, cysteine deprivation increased Fe^2+^ accumulation in both intracellular and mitochondrial compartments, and these increases were significantly attenuated by metformin and phenformin. Furthermore, rotenone or dissipation of the mitochondrial membrane potential with carbonyl cyanide m‐chlorophenyl hydrazone (CCCP) similarly attenuated mitochondrial Fe^2+^ accumulation under cysteine deprivation (Figure S3). These findings suggest that mitochondrial function contributes to Fe^2+^ accumulation during cysteine deprivation. However, the molecular mechanisms linking mitochondrial function to iron homeostasis during cysteine deprivation warrant further investigation.

Previous studies have demonstrated that mitochondria play an important role in cysteine deprivation‐induced ferroptosis. Our previous study demonstrated that mitochondrial ROS and VDAC1 oligomerization contribute to this process [25]. In addition, mitophagy‐mediated mitochondrial depletion has been reported to confer resistance to cysteine deprivation‐induced ferroptosis [2]. A previous study suggested that metformin and phenformin suppress mitochondrial permeability transition pore (MPTP) opening through inhibition of mitochondrial Complex I [34]. In the present study, pharmacological inhibition of MPTP opening with cyclosporine A significantly attenuated cysteine deprivation‐induced loss of cell viability and lipid peroxidation (Figure S4A, B). This protective effect was not accompanied by a reduction in cysteine deprivation‐induced MMP hyperpolarization. MMP was increased by cyclosporine A treatment alone, consistent with a previous report [35], and remained elevated during cysteine deprivation even in the presence of cyclosporine A (Figure S4C). This dissociation may reflect a low‐conductance, transient mode of MPTP opening that permits Ca^2+^ and ROS signaling without inducing a sustained collapse of mitochondrial membrane potential, in contrast to the classical high‐conductance opening associated with mitochondrial depolarization [36, 37, 38, 39]. These findings raise the possibility that MPTP‐related processes may contribute to ferroptotic progression through mechanisms other than global changes in mitochondrial membrane potential and may also be involved in the inhibitory effects of metformin and phenformin on cysteine deprivation‐induced ferroptosis. Further studies are required to clarify the precise role of MPTP in this process.

Under cysteine deprivation, pharmacological inhibition of ferroptosis, including iron chelation, restored cell viability, whereas the apoptosis inhibitor z‐VAD‐FMK had no effect. The necroptosis inhibitor necrostatin‐1 (Nec‐1) also significantly restored cell viability. These findings are consistent with previous reports showing that cystine deprivation is associated with both ferroptosis and necroptosis, but not apoptosis, in various cancer cells [40, 41]. Recent studies have further suggested functional crosstalk between ferroptosis and necroptotic signaling, with lipid peroxidation serving as a common mediator of both pathways [42, 43]. In this study, Nec‐1 also significantly reduced cysteine deprivation‐induced lipid peroxidation. Together, these findings suggest that cysteine deprivation‐induced cell death involves ferroptosis as well as necroptotic signaling. However, the involvement of necroptotic signaling was assessed independently of the biguanide experiments, and the effects of metformin and phenformin on this signaling pathway require further investigation.

The ferroptotic response may vary depending on the method used to induce cysteine deficiency. IKE, a potent erastin derivative and system Xc^−^ inhibitor, did not induce substantial cell death in H1299 cells, unlike direct cysteine deprivation (Figure S5). Previous studies have implicated α‐synuclein in erastin‐induced ferroptosis, whereas α‐synuclein expression has been reported to be low in lung adenocarcinoma and NSCLC cells [44, 45, 46]. Although the underlying mechanism remains unclear, such cell type‐specific factors may contribute to the limited response to IKE in H1299 cells. These observations suggest that the ferroptotic response to direct cysteine deprivation may differ from that induced by pharmacological inhibition of system Xc^−^.

## 5 Conclusion

In conclusion, metformin and phenformin attenuated cysteine deprivation‐induced ferroptosis in cancer cells. Our findings advance the mechanistic understanding of how biguanides modulate mitochondrial responses during cysteine deprivation‐induced ferroptosis. Given the increasing interest in ferroptosis‐based cancer therapies, the effects of biguanides should be carefully considered when developing cysteine deprivation‐based therapeutic strategies.

## Author Contributions

**Se‐Kyeong Jang:** data curation, formal analysis, investigation, methodology, validation, visualization, writing – original draft. **Selim Kim:** data curation, methodology, investigation. **Gyeongmi Kim:** methodology, data curation. **Jungil Hong:** methodology, resources. **In‐Chul Park:** conceptualization, supervision. **Hyeon‐Ok Jin:** writing – review and editing, supervision, project administration, funding acquisition.

## Funding

This work was supported by the Korea Institute of Radiological and Medical Sciences, 50531‐2026. National Research Foundation of Korea, RS‐2025‐00553853.

## Ethics Statement

The authors have nothing to report.

## Consent

The authors have nothing to report.

## Conflicts of Interest

The authors declare no conflicts of interest.

## Supporting information


**Figure S1:** Ferrostatin‐1 attenuates cysteine deprivation‐induced mitochondrial ROS accumulation and MMP hyperpolarization. (A, B) H1299 cells were treated with ferrostatin‐1 (Fer‐1) in complete or cysteine‐deprived medium. (A) Mitochondrial ROS levels were assessed by flow cytometry using MitoSOX. (B) Mitochondrial membrane potential (MMP) was assessed by flow cytometry using TMRE. Data are presented as bar graphs showing the mean fluorescence intensity relative to the control and are expressed as the mean ± SD (*n* = 3).
**Figure S2:** Effects of metformin on cell viability under cysteine deprivation in additional NSCLC cell lines. (A–D) A549 (A), H460 (B), H23 (C), and H820 (D) cells were treated with the indicated concentrations of metformin for 24 h in complete or cysteine‐deprived media. (A–D) Cell viability was assessed by MTT assay. (*n* = 3).
**Figure S3:** Rotenone and CCCP attenuate cysteine deprivation‐induced mitochondrial Fe2⁺ accumulation. (A) H1299 cells were treated with 200 nM rotenone for 12 h in complete or cysteine‐deprived medium. (B) H1299 cells were treated with 5 μM CCCP for 12 h in complete or cysteine‐deprived medium. (A, B) Mitochondrial Fe2⁺ levels were assessed by flow cytometry using Mito‐FerroGreen. Data are presented as bar graphs showing the mean fluorescence intensity (MFI) relative to the control and are expressed as the mean ± SD (*n* = 3; ***p < 0.001). These experiments were conducted independently of the core metformin/phenformin assays on separate experimental days using separately prepared cell cultures.
**Figure S4:** Cyclosporine A attenuates cysteine deprivation‐induced cell death. (A, B) H1299 cells were treated with 20 μM cyclosporine A for 24 h (A) or 12 h (B) in complete or cysteine‐deprived medium. (A) Cell viability was assessed by MTT assay and is presented as a percentage relative to the control. (B) Lipid peroxidation was assessed by flow cytometry using C11‐BODIPY. (C) Mitochondrial membrane potential (MMP) was assessed by flow cytometry using TMRE. Data are presented as the mean ± SD (*n* = 3; ***p < 0.001). These experiments were conducted independently of the core metformin/phenformin assays on separate experimental days using separately prepared cell cultures.
**Figure S5:** Effects of IKE and metformin on cell viability in H1299 cells. H1299 cells were treated with the indicated concentrations of imidazole ketone erastin (IKE) in the presence or absence of metformin for 24 h. Cell viability was assessed by MTT assay and is presented as a percentage relative to the untreated control (*n* = 3). IKE, imidazole ketone erastin.

## Data Availability

The data that support the findings of this study are available from the corresponding author upon reasonable request.
