# Supplementary material for: Metformin Inhibits Ferroptotic Cell Death by Suppressing Cysteine Deprivation‐Induced Mitochondrial Dysfunction
Source: Pharmacol Res Perspect. 2026 Oct 7;14(5):e70330. doi: 10.1002/prp2.70330 (PMC13646487; doi:10.1002/prp2.70330)
Supplement: Supplementary file 1 [file PRP2-14-e70330-s001.docx]

**Supplementary Figure 1.** Ferrostatin-1 attenuates cysteine deprivation-induced mitochondrial ROS accumulation and MMP hyperpolarization

(A, B) H1299 cells were treated with ferrostatin-1 (Fer-1) in complete or cysteine-deprived medium. (A) Mitochondrial ROS levels were assessed by flow cytometry using MitoSOX. (B) Mitochondrial membrane potential (MMP) was assessed by flow cytometry using TMRE. Data are presented as bar graphs showing the mean fluorescence intensity relative to the control and are expressed as the mean ± SD (n = 3).

**Supplementary Figure 2.** Effects of metformin on cell viability under cysteine deprivation in additional NSCLC cell lines

(A–D) A549 (A), H460 (B), H23 (C), and H820 (D) cells were treated with the indicated concentrations of metformin for 24 h in complete or cysteine-deprived media. (A–D) Cell viability was assessed by MTT assay. (n = 3)

**Supplementary Figure 3.** Rotenone and CCCP attenuate cysteine deprivation-induced mitochondrial Fe²⁺ accumulation

(A) H1299 cells were treated with 200 nM rotenone for 12 h in complete or cysteine-deprived medium. (B) H1299 cells were treated with 5 μM CCCP for 12 h in complete or cysteine-deprived medium. (A, B) Mitochondrial Fe²⁺ levels were assessed by flow cytometry using Mito-FerroGreen. Data are presented as bar graphs showing the mean fluorescence intensity (MFI) relative to the control and are expressed as the mean ± SD (n = 3; ***p < 0.001). These experiments were conducted independently of the core metformin/phenformin assays on separate experimental days using separately prepared cell cultures.

**Supplementary Figure 4.** Cyclosporine A attenuates cysteine deprivation-induced cell death

(A, B) H1299 cells were treated with 20 μM cyclosporine A for 24 h (A) or 12 h (B) in complete or cysteine-deprived medium. (A) Cell viability was assessed by MTT assay and is presented as a percentage relative to the control. (B) Lipid peroxidation was assessed by flow cytometry using C11-BODIPY. (C) Mitochondrial membrane potential (MMP) was assessed by flow cytometry using TMRE. Data are presented as the mean ± SD (n = 3; ***p < 0.001). These experiments were conducted independently of the core metformin/phenformin assays on separate experimental days using separately prepared cell cultures.

**Supplementary Figure 5.** Effects of IKE and metformin on cell viability in H1299 cells

H1299 cells were treated with the indicated concentrations of imidazole ketone erastin (IKE) in the presence or absence of metformin for 24 h. Cell viability was assessed by MTT assay and is presented as a percentage relative to the untreated control (n = 3). IKE, imidazole ketone erastin.
